# Serum IgG Antibodies from Pregnant Women Reacting to Mimotopes of Simian Virus 40 Large T Antigen, the Viral Oncoprotein

**DOI:** 10.3389/fimmu.2017.00411

**Published:** 2017-04-10

**Authors:** Elisa Mazzoni, Mariantonietta Di Stefano, Josè R. Fiore, Federica Destro, Marco Manfrini, John Charles Rotondo, Maria V. Casali, Fortunato Vesce, Pantaleo Greco, Gennaro Scutiero, Fernanda Martini, Mauro G. Tognon

**Affiliations:** ^1^Department of Morphology, Surgery and Experimental Medicine, Section of Pathology, Oncology and Experimental Biology, Laboratories of Cell Biology and Molecular Genetics, University of Ferrara, Ferrara, Italy; ^2^Department of Clinical and Experimental Medicine, Clinic of Infectious Diseases, School of Medicine, University of Foggia, Foggia, Italy; ^3^Hospital Headquarter Department, State Hospital, Institute for Social Security, Borgo Maggiore, San Marino; ^4^Department of Morphology, Surgery and Experimental Medicine, Section of Obstetrics and Gynecology, University of Ferrara, Ferrara, Italy

**Keywords:** pregnancy, polyomavirus, Simian virus 40, infection, antibody, seroprevalence, oncogene, ELISA

## Abstract

Simian virus 40 (SV40) *large T antigen* (*LT*) coding sequences were revealed in different human samples, whereas SV40 antibodies (Ab) were detected in human sera of cancer patients and healthy individuals, although with a lower prevalence. Previous studies carried out by the neutralization assay gave a SV40 seroprevalence, in the general population, up to 8%, although higher rates, 12%, were detected in kidney transplant children, in a group of HIV-positive patients, and in healthy females. In this study, serum samples from pregnant women, together with those from non-pregnant women, were analyzed to check the prevalence of IgG Ab reacting to SV40 LT antigens. Serum samples were collected from pregnant and non-pregnant women, with the same mean age. Women were in the range of 15–48 years old. Samples were assayed by an indirect ELISA employing specific SV40 LT mimotopes as antigens, whereas functional analysis was performed by neutralization of the viral infectivity in cell cultures. As a control, sera were analyzed for Ab against BK polyomavirus (BKPyV), which is a human polyomavirus homologous to SV40. Statistical analyses employed chi-square with Yates’ correction, and Student’s *t* tests. Indirect ELISAs indicated that pregnant women tested SV40 LT-positive with a prevalence of 17% (23/134), whereas non-pregnant women had a prevalence of 20% (36/180) (*P* > 0.05). Ab against BKPyV were detected with a prevalence of 80% in pregnant women and with a prevalence of 78% in non-pregnant women. These data indicate that SV40 infects at a low prevalence pregnant women. We may speculate that SV40, or a close human polyomavirus still undetected, could be transmitted from mother to fetus.

## Introduction

Simian virus 40 (SV40) was detected in early poliovirus vaccines. SV40 was present as a natural infectious agent in kidney cells, from wild macaques, employed for the production of anti-polio vaccines. Millions of individuals between 1955 and 1963 were vaccinated with anti-polio vaccines with SV40 as contaminant. These vaccines, as well as other SV40-contaminated vaccines against hepatitis A virus, respiratory syncytial virus, and adenovirus, were sources of SV40 exposure for the human population worldwide (1, 2). SV40 transforms *in vitro* cells of different types, including human cells, whereas *in vivo* it induces multiple tumors in experimental animals (1, 3, 4). SV40 DNA sequences were detected in different normal specimens from healthy subjects (5–10). Moreover, SV40 sequences were revealed by molecular biology techniques, in human cancers of distinct histotypes, such as brain and bone tumors, mesothelioma, different lymphoproliferative disorders, including non-Hodgkin lymphoma. Among SV40-positive tumors, brain tumors as ependymomas and choroid plexus papillomas, as well as osteosarcomas mainly affect children and adolescents (2). The association between pediatric tumors and SV40 suggests that the viral infection could be acquired during the pregnancy period or soon after the delivery of the offspring. It should be recalled that these human tumors correspond to the neoplasms that are induced by SV40 in experimentally inoculated rodents (11) or in transgenic mice with SV40 Tag under tissue-specific promoter–enhancer (12, 13).

The World Health Organization/International Agency for Research on Cancer indicated that there is not enough firm evidence to classify SV40 as a carcinogenic viral agent of humans (14). Although SV40 infections have been documented in certain populations in different geographic regions, more studies are needed to investigate the prevalence of SV40 in humans and the natural history of this infection.

Seroprevalence surveys are a common approach to examine the distribution of a viral infection within a host population. The neutralization assay, which is considered in the field the most efficient and specific technique to detect SV40 antibodies (Ab) in human sera, was employed in several investigations. This method in US investigations gave a seroprevalence of SV40 Ab up to 8%, whereas in kidney transplant pediatric patients, HIV patients, and women with a Hispanic genetic background, a higher prevalence was detected (15). In Italy, SV40 prevalence was found, with the same technical approach, to be higher in the range of 12% (16). However, this technical approach has several disadvantages: it is expensive, lengthy, and because of the many different methodological tasks, it requires specialized trained personal. The neutralization assay disadvantages do not allow its use in serological surveys with a large sample size. SV40 Ab were detected using enzyme immunoassays (EIA) with SV40 antigens represented by virus-like particles (VLPs) or soluble VP1 capsid protein, as recombinant products. In EIA reactions, all VP Ab are detected, including non-neutralizing ones and those that recognize cross-reacting antigens with other highly homologous polyomaviruses, such as BK virus (BKPyV) and JC virus (JCPyV). The cross-reactivity is the major limitation of this approach because it gives non-specific reactivity for SV40 (17–22).

Novel indirect ELISAs with specific SV40 mimotopes, as synthetic peptides, representing viral capsid proteins VP 1–3 (23) and viral LT oncoprotein (24, 25) seems to circumvent these problems, i.e., the cross-reactivity among closely related polyomaviruses. Recent studies with these novel ELISAs documented SV40 Ab in healthy subjects with an estimated seroprevalence of 18–20%.

Molecular biology and immunological investigations reported on the presence of SV40 footprints in samples from healthy subjects and patients who had not administered with SV40-contaminated vaccines (24, 26). These studies indicate that the human-to-human contagion could be responsible of the SV40 infection in the human population. At present, the prevalence of SV40 spread is much lower, about 18%, than that detected for other ubiquitous polyomaviruses, such as BKPyV, JCPyV, and Merkel cell polyomavirus (MCPyV), which is in the range of 80% (14, 23, 25).

In previous investigations, IgG serum Ab reacting with SV40 VP mimotopes and with neutralization activity were reported in pregnant women and non-pregnant women. Herein, we report new data from the investigation, which determines the prevalence of Ab against SV40 LT, the viral oncoprotein, in pregnant women using a novel indirect ELISA with two synthetic peptides corresponding to SV40 LT mimotopes.

## Materials and Methods

### Study Design and Setting

Samples were from our serum collections (23, 25, 27, 28). They were collected from pregnant women (*n* = 134) and non-pregnant women (*n* = 180) with a similar age. The mean age was 32 years (15–48 years). All serum samples (*n* = 314) were assayed by an indirect ELISA with mimotopes, represented by specific synthetic peptides, from SV40 large T antigen (LT).

Study participants provided written informed consent at the time of the hospital admission. Samples were collected from discarded sera, after routine clinical laboratory analyses, before their incineration. The Ethic Committee of Ferrara, approved the study, number 151078, on April 14, 2016.

### Indirect ELISA

The indirect ELISA employed herein has been recently published (24, 25).

#### Peptide Design

Computer-assisted analyses enabled us to select two specific SV40 LT peptides, known as A and D, respectively, which are encoded by the viral LT early gene. SV40 Tag mimotopes were compared with LT amino acids (a.a.) from human BKPyV and JCPyV polyomaviruses, which are highly homologous to SV40, as well as with other, less homologous, polyomaviruses.^1^ The a.a. residues of the two specific SV40 LT peptides showed low homology with BKPyV and JCPyV Tag. Preliminary ELISA results indicated that the two selected peptides did not cross-react with BKPyV and JCPyV hyperimmune sera employed as controls. The two A and D peptides map on the LT oncoprotein,^2^ as shown earlier (24, 25). The a.a. sequences of the two LT A and D peptides are as follows:

LT A: NH2-G S F Q A P Q S S Q S V H D H N Q P Y H I-COOH (Tag a.a. 669–689).
LT D: NH2-H E T G I D S Q S Q G S F Q A P Q S S Q S V H D-COOH (Tag a.a. 659–682).

Mimotopes A and D overlap from Tag a.a.669 to 682. SV40 LT synthetic peptides A and D were employed in indirect ELISAs (see below) because reacting specifically with a hyperimmune serum of a rabbit immunized with the purified SV40 LT protein (positive control sera) and with SV40 VP-positive human sera, tested earlier (23). SV40 LT A and D synthetic peptides did not react with BKPyV and JCPyV hyperimmune sera, employed as negative control. A human negative peptide, used as control, was the neuropeptide S (hNPS), a.a. sequence SFRNGVGTGMKKTSFQRAKS. This human neuropeptide is non-liked to SV40 (25). The synthetic peptides were from the University of Ferrara firm, the UFPeptides s.r.l., Ferrara, Italy.

#### Peptide Coating

Briefly, plates coated with synthetic peptides were used to reveal specific SV40 LT Ab in human serum samples. These SV40 LT A and D mimotopes are from the a.a. residues 669–689 (21 a.a.) and 659–682 (24 a.a.), respectively. ELISA plates (Nunc-immuno plate PolySorp, CelBio, Milan) were coated with 5 µg of the mimotope A or D, diluted in 100 µl of Coating Buffer 1×, pH 9.6 (Candor Bioscience, Wangen, Germany). Plates were placed at 4°C for 16 h.

#### Blocking Phase

To remove unattached peptide, plates were rinsed three times with Washing Buffer (Candor Bioscience, Wangen, Germany). Blocking was performed with 200 μl/well of blocking solution containing casein (Candor Bioscience, Wangen, Germany) at 37°C for 90 min.

#### Primary Antibody

Then, plates were rinsed three times with Washing Buffer (Candor Bioscience, Wangen, Germany) using washing apparatus (Thermo Electron Corp., model Well wash 4MK2, Vantaa, Finland). Different wells were covered with 100 µl containing the following sera: a positive control represented by the immune rabbit serum containing anti-SV40 LT Ab; the negative controls were immune sera with anti-BKPyV and anti-JCPyV Ab and three human serum samples tested SV40-negative in earlier studies (24, 25). Human serum samples from pregnant women and non-pregnant women were diluted in Low Cross-Buffer pH 7.2 (Candor Bioscience, Wangen, Germany) at 1:20 and were added to the plate. Additional controls in each plate included a well with the secondary antibody only and other wells void of both primary and secondary Ab. The plate was incubated at 37°C for 90 min.

#### Secondary Antibody Addition

After 90 min of incubation, a triple rinsing cycle was repeated and then the secondary antibody solution was added to each well. The solution contained goat anti-human or goat anti-rabbit IgG heavy (H) and light (L) chain-specific peroxidase conjugate (Calbiochem-Merck, Darmstadt, Germany) diluted 1:10,000 in Low Cross-Buffer (Candor Bioscience, Wangen, Germany). The reaction mixture was incubated at room temperature for 90 min.

#### Optical Density (OD) Reading

At the end of the incubation period, the plates were rinsed three times with washing buffer and then treated with 100 µl of 2,2′-azino-bis 3-ethylbenzthiazoline-6-sulfonic acid (ABTS) solution (Sigma-Aldrich, Milan, Italy), which reacted with the peroxidase enzyme to yield the color reaction. The plate was then read spectrophotometrically (Thermo Electron Corp., model Multiskan EX, Vantaa, Finland) at a wavelength (λ) of 405 nm. This OD reading reflected the extent of immune complexes formed by the presence of specific Ab, which bound to the SV40 LT synthetic peptide/epitopes/mimotopes.

#### Cutoff Determination

Cutoff values were determined for each assay using the OD reading of the three negative control sera, which were added to the SD and multiplied three times (+3 SD). The three SV40-negative control sera were selected from those below the cutoff value determined with second-degree polynomial regression by plotting the ranked net OD individual values for each peptide. The OD representation was obtained from a second-degree polynomial regression for LT A and D mimotopes, as reported before for BKPyV and MCPyV VLPs (24, 25). Our representations show an inflection point for peptide A and peptide D at 0.19–0.18, respectively. Immune serum samples were considered SV40 LT-positive when reacting to both peptides A and D, in three replica ELISA experiments carried out by three different operators.

### Hemagglutination (HA) Assay and Hemagglutination Inhibition (HAI) Assay

In order to verify which sera from pregnant women were BKPyV-positive, HAI assays were carried out, as described before (23, 29). Briefly, BKPyV viral working stocks, employed for HAI, were obtained in infected Vero cells, purchased from the ATCC. BKPyV viral titer, determined by the HA assay was 1.6 × 10^2^ hemagglutinating units, corresponding to 1.6 × 10^6^ plaque forming unit (PFU)/mL. BKPyV was employed as antigens in HA and HAI tests. HA and HAI titers for BKPyV were obtained as reported before (23, 29, 30). These assays allow the detection of serum Ab against the human polyomavirus BKPyV, which abolish its agglutination properties. The HA titer is the highest viral dilution that still hemagglutinates the human herytrocytes, whereas the HAI titer is the highest dilution of the immune serum under analysis that still inhibits completely the viral HA.

### SV40 Neutralization Assay

CV-1 monkey kidney cells, which are cells permissive to SV40 infection, were employed for the neutralization assay *in vitro*. The SV40 infectivity was scored as a reduction of the number of PFUs in CV-1 monolayers. The neutralization of the SV40 infectivity was assayed by mixing the human serum under analysis, diluted at 1:20 in phosphate buffered saline (PBS), with 5 × 10^4^ PFUs of SV40; then, the solution was incubated at 37°C for 30 min. The solution was layered on the CV-1 monolayer for 2 h at 37°C. Then, the inoculum was discarded from the cells and the monolayer rinsed three times with DMEM. Each sample was analyzed twice. The controls were as follows: (i) CV-1 with PBS; (ii) SV40 with PBS; (iii) SV40 with an immune human sample, the positive control; (iv) SV40 with a non-immune serum, the negative control. The appearance of the SV40 cytopathic effect (CPE) had inspected in CV-1 infected monolayers by a light microscope for 21 days. The neutralization assay was performed in triplicate, while the neutralizing activity of the serum under analysis was checked by the inhibition of SV40 CPE.

### Statistical Analysis

The prevalence of SV40-positive serum samples from pregnant and non-pregnant women was determined using chi-square with Yates’ correction. The serologic profile of serum antibody reactivity to SV40 LT mimotopes was statistically analyzed using the Student’s *t* tests. All computational analyses were performed with Prism 6.0 (GraphPad software, San Diego, CA, USA). For all tests, *P* was considered to be statistically significant when *P* < 0.05.

## Results

### IgG Ab Reacting to SV40 LT Mimotopes in Serum Samples from Pregnant Women

**Figure 1:**
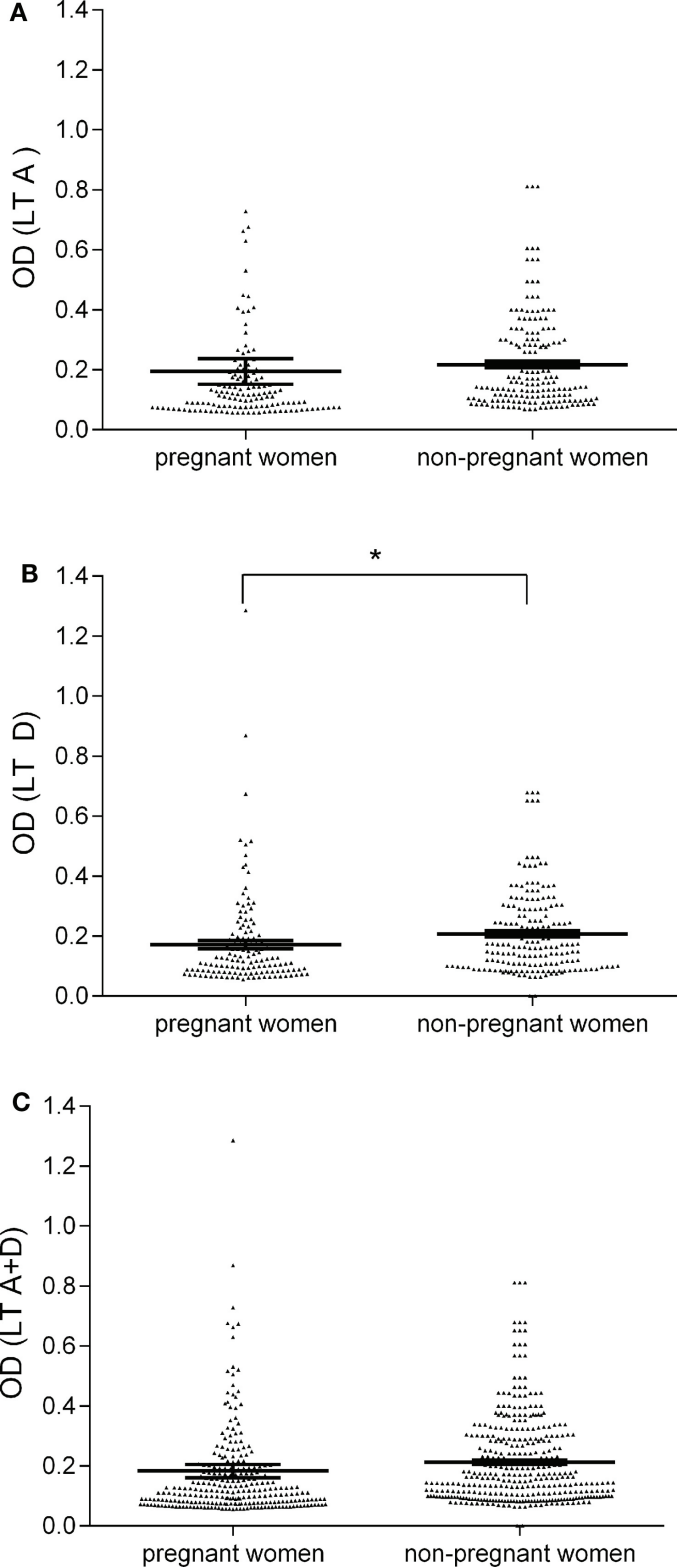
**Serologic profile of serum antibody reactivity to Simian virus 40 (SV40) LT mimotopes**. Immunologic data are from total samples of pregnant and non-pregnant women. Results are presented as optical density (OD) values at λ 405 nm for serum samples diluted 1:20 and assayed using indirect ELISAs. In the scatter dot plotting, each plot represents the dispersion of individual sample OD value to a mean level, indicated by the long horizontal line inside the scatter with SEM (31) marked by a short horizontal line for each age group. Data were analyzed with unpaired *t* test (OD mean, 95% CI). **(A)** OD values of antibodies (Ab) against SV40 mimotope LT A in pregnant women (0.19 OD, 95% CI = 0.11–0.28) was similar to that observed in non-pregnant women (0.22 OD, 95% CI = 0.19–0.24, *P* > 0.05). **(B)** The mean OD of sera against SV40 mimotope LT D in pregnant women (0.17, 95% CI = 0.14–0.19) was lower than that detected in non-pregnant women (0.20, 95% CI = 0.19–0.23, *P < 0.05). **(C)** OD values of Ab against SV40 mimotopes, both peptides LT A and LT D, found in pregnant women (0.18 OD, 95% CI = 0.14–0.23) were similar to those observed in non-pregnant women (0.21 OD, 95% CI = 0.20–0.23, *P* > 0.05).

Sera from pregnant women (*n* = 134) and non-pregnanat women (*n* = 180) were tested by indirect ELISAs for IgG Ab reactive to SV40 LT A and D synthetic peptides (Table 1). Pregnant women sera, diluted to 1/20, were assayed for their ability to react to SV40 LT A mimotope. Pregnant women samples tested positive for LT peptide A were 32 out of 134, with a prevalence of 24% (Table 1). ODs for SV40 LT-positive sera were in the range 0.19–0.73 (Figure 1). Then, sera were assayed for the other SV40 LT-specific peptide, the D mimotope. Samples reacted with the LT D peptide the similar prevalence, 23% (31/134) (Table 1) as it was detected for the LT A peptide. ODs range was 0.18–1.28 (Figure 1). Serologic profiles are shown in Figure 1.

**Table 1:** **Prevalence of immunoglobulin G antibodies reacting Simian virus 40 (SV40) LT mimotopes in serum samples from pregnant and non-pregnant women**.

Subject	Number of samples	Mean age (range) (years)	Number of positive samples (%)		
			LT A	LT D	LT A + D
Pregnant women	134	32 (15–48)	32 (24)	31 (23)	23 (17)
Non-pregnant women	180	30 (18–40)	48 (27)	51 (28)	36 (20)

*Human sera were from pregnant and non-pregnant women. No statistically significant differences of SV40 seroprevalence were detected between the two cohorts (*P* > 0.05). Statistical analysis was performed using the chi-square test with Yates’ correction*.

The human peptide hNPS, the negative control, did not react with human sera under analysis. OD values were in the range of 0.050–0.095, an OD reading which is expected for human sera tested SV40-negative. The hyperimmune rabbit serum with Ab against SV40 LT had OD readings of 2.9 and 2.2 for peptides A and D, respectively. In our indirect ELISAs, the OD value of 0.18 is the cutoff for the SV40 LT-negative samples, with an OD < 0.18, and SV40 LT-positive samples, with an OD value >0.18. Control sera were six SV40 LT-positive human sera, which reacted with SV40 VP mimotopes in previous investigations, together with a hyperimmune serum of a rabbit immunized with purified SV40 LT protein. Herein, human sera tested SV40 LT-positive had OD values up to 1.286, whereas human sera tested SV40 LT-negative had OD less than 0.18 (Figure 1). In our study, only those sera (*n* = 23) which tested positive for both SV40 LT mimotopes A and D, with an overall prevalence of 17%, were considered SV40 LT-positive (Table 1). The majority of serum samples (*n* = 102/134; 76%) that were found to be negative for the LT A peptide did not react to SV40 LT D antigen. The different prevalence of LT A-positive sera (32/134; 24%) and LT D-positive sera (31/134; 23%) was not significant (*P* > 0.05). The prevalence of 24 and 23% detected for the single peptide A and peptide D, respectively, did not differ statistically from the prevalence of 17% (23/134) detected for samples found to be SV40-positive for both peptides A + D. In our indirect ELISA, SV40-positive sera had general spectrophotometric readings in the range of 0.18–1.28 OD.

Sera of non-pregnant women were analyzed by the same indirect ELISA with the SV40 LT mimotopes A and D. Samples of non-pregnant women had a prevalence of specific SV40 LT Ab of 20% (36/180) (Table 1).

### HAI Test for the Presence of Neutralizing Ab against BKV

The presence of BKPyV serum Ab in pregnant and non-pregnant women was determined using the HAI assay. Specifically, *n* = 30 and *n* = 50 sera of pregnant women and non-pregnant women, diluted 1/20 in PBS, were incubated with BKPyV (8 HAU) virions for 1 h (30). Then, sera added to virions were assayed with human red cells, group 0, Rh+, for 5 h at +4°C. Among sera, 24 from pregnant women out of 30 (80%) and 39 from 50 of the non-pregnant group (78%) inhibited the hemagglutination property of BKPyV (Table 2). The reproducibility of the serological results was assessed in three replica experiments carried out by independent operators with no data variability. Altogether, these assays allowed us to verify, which serum samples were SV40-positive only.

**Table 2:** **Prevalence of antibodies (Ab) against BKPyV using hemagglutination inhibition assay**.

Subject	BKPyV Ab positive^a^/analyzed sera (%)
Pregnant women	24/30 (80)
Non-pregnant women	39/50 (78)

*The prevalence of Ab against BKPyV in sera samples of pregnant women was compared to that of non-pregnant women. Statistical analysis was performed with the chi-square test with Yates’ correction. No statistically significant differences of BKPyV seroprevalence, between the two cohorts (*P* > 0.05)*.

*^a^BKPyV Ab-positive sera were at 1:20 dilution*.

### Neutralization of SV40 Infectivity in Permissive Cells by Serum Ab from Pregnant Women

**Figure 2:**
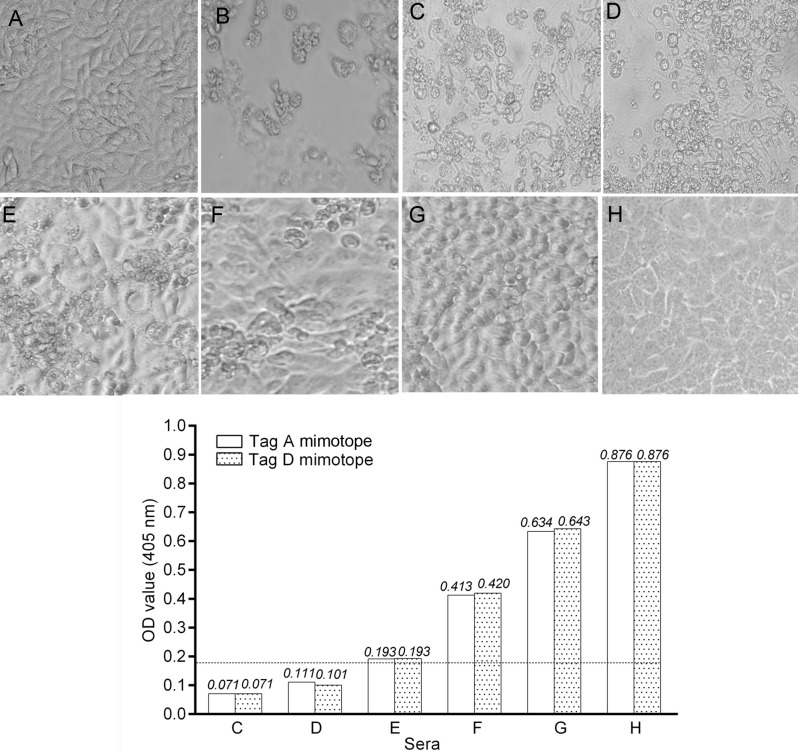
**Inhibition of Simian virus 40 (SV40) cytopathic effect (CPE) in CV-1 infected cells by human immune sera**. Inhibition of SV40 CPE in infected cells by human serum samples from pregnant women and non-pregnant women. Upper panel. **(A)** Negative control represented by uninfected CV-1 cells. **(B)** Positive control represented by the CPE induced by SV40 in CV-1 infected cells. **(C,D)** These sera did not inhibit SV40 CPE. **(E–G)** SV40-positive sera inhibited SV40 CPE with different degrees. **(H)** This serum sample completely inhibited SV40 CPE. Sample H had an optical density (OD) of 0.876 for the LT mimotopes. Bottom panel: there was a correlation between the OD values and CPE inhibition activity of tested immune sera. Indeed, serum samples C and D that were under the cutoff value did not inhibited SV40 CPE, while sera E, F, and G with an OD in the range of 0.193–0.643 inhibited partially with a different degree SV40 CPE. The serum in H panel, with the higher OD value, inhibited completely SV40 CPE.

To verify the neutralization activity of SV40 immune sera from pregnant women, an inhibition test was performed (29). SV40 immune sera (*n* = 4) from pregnant women and non-pregnant women (*n* = 4) with an OD (range OD = 0.193–0.876) were challenged to inhibit the SV40 CPE in CV1-infected monolayers. Together with SV40-positive sera, two additional SV40-negative sera (range OD = 0.071–0.111) were added to the neutralization assay, as control. These sera were BKPyV negative, as determined by HAI. SV40 CPE was hampered or abolished indicating that tested sera carried neutralizing Ab against SV40 (Figure 2). This result strongly suggests that a SV40 infection occurred in pregnant women, tested SV40-positive, whereas the neutralizing Ab present in those immune sera were elicited by SV40 and not by the homolog BKPyV (30).

## Discussion

### Principal Findings of the Study

This is the first study providing SV40 LT seroprevalence data for pregnant and non-pregnant women, by indirect ELISAs with specific LT mimotopes. SV40 infection has been studied in healthy individuals of different age, such as children, adults and elderly (20, 32–37) and in patients with different cancers found to be linked to SV40 (38–43).

This study reports an overall prevalence of 17% in pregnant women tested seropositive for SV40 LT Ab (Table 1). These findings substantiate previous observations of SV40 LT Ab in different age cohorts of normal individuals (24, 25). The overall prevalence of SV40 LT IgG Ab (17%) detected in pregnant women using indirect ELISAs with mimotopes was similar to that reported by previous studies carried out by neutralization assays (10.3%) (16) and indirect ELISA with SV40 viral capsid protein (VP) mimotopes (13%) (29). The difference among these studies is significant because in the present survey, carried out in part with new sera from pregnant women and non-pregnant women (this study), SV40 LT was the target of the investigation. Indeed, SV40 LT, the viral oncoprotein, is responsible of the tumorigenic activity *in vivo* of this small DNA tumor virus. The novelty of this investigation can be appreciated if the potential viral oncogenic activity of SV40 LT is considered (44), together with the viral DNA replication activity driven by the LT (45). SV40 seems to multiply in pregnant women with the consequence that SV40 may exert its tumorigenic potential in some subjects. The previous reports, with immunological data of SV40 viral capsid proteins, indicated that SV40 infects humans and that the structural capsid proteins elicited specific IgG Ab. The detection of LT Ab in pregnant women indicates a potential risk of tumorigenesis for some of them and their embryo/fetus/newborn. At the same time, this study on the IgG SV40 LT confirms and extends the immunological data on SV40 reported in previous investigations.

Taken together, these results provide a comprehensive analysis of SV40 seroprevalence during pregnancy. The pregnant women analyzed herein were too young to be contaminated by SV40 with the early anti-polio vaccines. Indeed, contaminated poliovaccines were supposedly SV40 free after 1963, it is unlikely that vaccine exposure explains the SV40 antibody positivity of our cohorts (29). These data together with previous results indicate that other sources of SV40 exposure must exist in the human population. Very likely, the SV40 reservoir is the human host, whereas the infection may reflect the human-to-human contagion. Interestingly, polyomaviruses BKPyV, JCPyV, and SV40 have been detected in stool and tonsil samples of children and adults indicating that these human anatomical sites/specimens could carry these viral agents. In turn, infections could occur in the family settings and could spread from mother to child, as well. We observed a low SV40 antibody prevalence in sera of pregnant women, even if there was no statistically significant difference from the prevalence found in non-pregnant women. This low SV40 prevalence could be due to the pregnancy status. Indeed, the pregnancy is a well known condition, which leads to the immune tolerance effect. In addition, in pregnant women the increased volume of serum may be responsible of the IgG dilution, which in turn diminished the antibody titer. It is also possible that SV40 poorly multiplies in human cells, which are only semipermissive to this virus, thus affecting SV40 Ab response over time. The transient immune depression/immune tolerance/IgG dilution associated with pregnancy could allow reactivation of latent viruses, including polyomaviruses. In this instance, it would be possible that the SV40 transplacental transmission from mother to fetus may occur, as demonstrated for BKPyV (46), a closely SV40 related human polyomavirus.

### BKPyV Seroprevalence

The prevalence of BKPyV Ab, although tested herein in a limited sample size, was approximately 80%, both in pregnant and non-pregnant women. These data indicate that the pregnancy status does not alter the immune response against the human polyomavirus BKPyV.

Transplacental transmission of polyomaviruses can occur in animals, including the murine polyomavirus in mice (47, 48), rats (49), and SV40 in hamsters (50, 51). It has been reported that BKPyV, which is closely related to SV40, can cross the human placenta during pregnancy. Indeed, BKPyV has been detected in fetal organs, such as kidney and brain, which are tissues were BKPyV remains in the latent state after the primary infection (46).

### SV40 Spread in Pregnant Women

Simian virus 40 infection detected in pregnant women should be taken into consideration because of its oncogenic potential. Indeed, the immune tolerance could account in some pregnant women for the absence/a lower specific immune reactivity against this small DNA tumor virus. SV40 after its multiplication/reactivation in pregnant woman may cross the placenta barrier thus infecting the embryo/fetus. If this event occurs, SV40 because of its oncogenic potential could be responsible of ependymoma and choroid plexus papilloma onset, which are pediatric brain tumors arising in children 6 months–2 years old (52, 53). It should be recalled that, in previous investigations these human cancers, together with glioblastoma multiforme (29, 54, 55) tested SV40-positive at high prevalence (6, 38, 56). In conclusion, SV40 LT Ab revealed in sera of pregnant women suggest further that SV40, or a closely related unknown polyomavirus, infects normal subjects many decades after the administration of vaccines contaminated by SV40. This result, together with previous data, indicates that SV40 is now spreading in humans by a person-to-person contagion. The health risk due to SV40 infection in normal individuals remains to be elucidated. The clinical profile of SV40 infection during pregnancy, while requiring additional investigations, is of interest because SV40 could be a potential risk for the onset of specific cancers found to be associated to the this oncogenic virus.

## Ethics Statement

This study was carried out in accordance with the recommendations of the County Ethics Committee of Ferrara. The written informed consent from all subjects was obtained at the time of the hospital admission. All subjects gave written informed consent in accordance with the Declaration of Helsinki. The protocol number 151078 was approved by the County Ethics Committee of Ferrara on April 14, 2016.

## Author Notes

EM was a fellowship recipient of the Fondazione Umberto Veronesi, Milan.

## Author Contributions

MT, FM, and EM designed research; FD, JR, and MM carried out the experiments; MS, JF, MC, FV, PG, and GS analyzed data and arranged clinical aspect; EM, FM, and MT wrote the paper.

## Conflict of Interest Statement

The authors declare that the research was conducted in the absence of any commercial or financial relationships that could be construed as a potential conflict of interest.

## Footnotes

^1^http://blast.ncbi.nlm.nih.gov.

^2^http://www.ncbi.nlm.nih.gov/nuccore.
