# Corrigendum: Serum IgG antibodies from pregnant women reacting to mimotopes of Simian virus 40 large T antigen, the viral oncoprotein

**DOI:** 10.3389/fimmu.2023.1329317

**Published:** 2024-01-05

**Authors:** Elisa Mazzoni, Mariantonietta Di Stefano, Josè R. Fiore, Federica Destro, Marco Manfrini, John Charles Rotondo, Maria V. Casali, Fortunato Vesce, Pantaleo Greco, Gennaro Scutiero, Fernanda Martini, Mauro G. Tognon

**Affiliations:** ^1^ Department of Morphology, Surgery and Experimental Medicine, Section of Pathology, Oncology and Experimental Biology, Laboratories of Cell Biology and Molecular Genetics, University of Ferrara, Ferrara, Italy; ^2^ Department of Clinical and Experimental Medicine, Clinic of Infectious Diseases, School of Medicine, University of Foggia, Foggia, Italy; ^3^ Hospital Headquarter Department, State Hospital, Institute for Social Security, Borgo Maggiore, San Marino; ^4^ Department of Morphology, Surgery and Experimental Medicine, Section of Obstetrics and Gynecology, University of Ferrara, Ferrara, Italy

**Keywords:** pregnancy, polyomavirus, Simian virus 40, infection, antibody, seroprevalence, oncogene, ELISA

In the published article, there was an error in **Figure 2** as published. Some technical problems may have occurred regarding the **Figures 2C, D** panels. There was an overlapping of two photographs taken at the same time, causing a partial duplication of the same field. The corrected **Figure 2** and its caption appear below.

**Figure 2:**
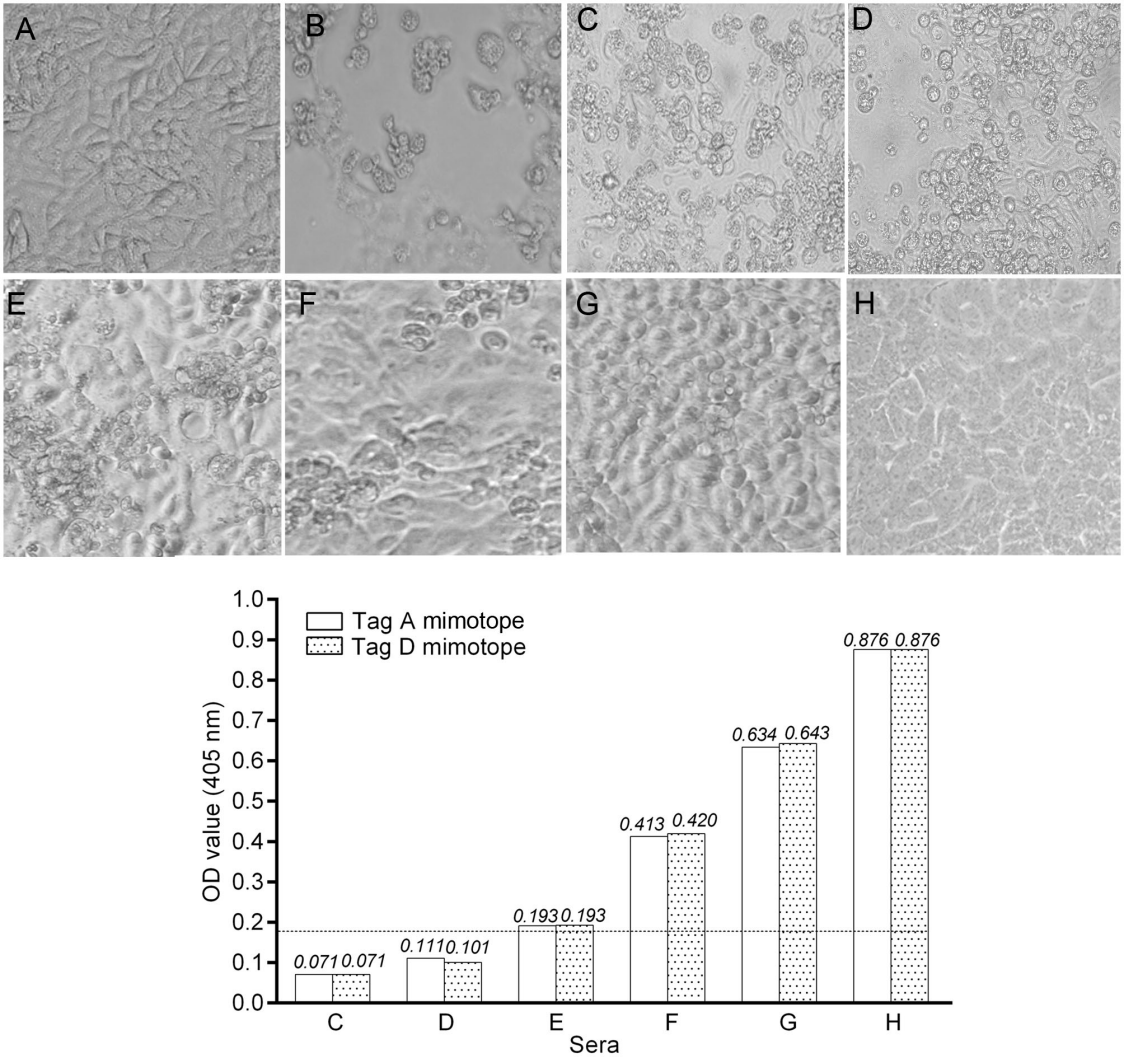
Inhibition of Simian virus 40 (SV40) cytopathic effect (CPE) in CV-1 infected cells by human immune sera. Inhibition of SV40 CPE in infected cells by human serum samples from pregnant women and non-pregnant women. Upper panel. **(A)** Negative control represented by uninfected CV-1 cells. **(B)** Positive control represented by the CPE induced by SV40 in CV-1 infected cells. **(C, D)** These sera did not inhibit SV40 CPE. **(E–G)** SV40-positive sera inhibited SV40 CPE with different degrees. **(H)** This serum sample completely inhibited SV40 CPE. Sample H had an optical density (OD) of 0.876 for the LT mimotopes. Bottom panel: there was a correlation between the OD values and CPE inhibition activity of tested immune sera. Indeed, serum samples C and D that were under the cutoff value did not inhibited SV40 CPE, while sera E, F, and G with an OD in the range of 0.193–0.643 inhibited partially with a different degree SV40 CPE. The serum in H panel, with the higher OD value, inhibited completely SV40 CPE.

The authors apologize for this error and state that this does not change the scientific conclusions of the article in any way. The original article has been updated.

